# S2-4 Physical literacy in Denmark: from concept to intervention

**DOI:** 10.1093/eurpub/ckad133.012

**Published:** 2023-09-11

**Authors:** Peter Elsborg, Paulina S Melby, Mette Kurtzhals, Knud Ryom, Glen Nielsen, Peter Bentsen

**Affiliations:** Copenhagen University Hospital – Bispebjerg and Frederiksberg, Center for Clinical Research and Prevention, Denmark; Copenhagen University Hospital – Bispebjerg and Frederiksberg, Center for Clinical Research and Prevention, Denmark; Department of Sports Science and Clinical Biomechanics, University of Southern Denmark, Denmark; Copenhagen University Hospital – Bispebjerg and Frederiksberg, Center for Clinical Research and Prevention, Denmark; Department of Nutrition, Exercise and Sports, University of Copenhagen, Denmark; Department of Public Health, Aarhus University, Denmark; Department of Nutrition, Exercise and Sports, University of Copenhagen, Denmark; Copenhagen University Hospital – Bispebjerg and Frederiksberg, Center for Clinical Research and Prevention, Denmark; Department of Geosciences and Natural Resource Management, University of Copenhagen, Denmark

## Abstract

**Purpose:**

Physical literacy (PL) is a holistic concept describing individuals’ prerequisites for engaging in physical activities through the life course. Interest in research on PL has increased rapidly in Denmark in the recent years. Researchers from the three largest universities, the largest university college, as well as research centers at two regional university hospitals have initiated projects with either PL as their core subject or using PL as a guiding theoretical framework for or as an outcome of physical activity interventions. These research projects have a conceptual purpose (Elsborg, Ryom, et al., 2021), a methodological purpose such as development of assessment tools (Elsborg, Melby, et al., 2021), and a purpose of investigating health-related correlates (Elsborg, Heinze, et al., 2021; Melby et al., 2021). Furthermore, interventional research projects that focus on how PL development is best stimulated in different age groups and target groups have recently begun (Ryom et al., 2021; Elsborg et al., 2021). The researchers that have been inspired by PL and are currently conducting PL research in Denmark come mainly from a motivational / sport and exercise psychological and/or public health background and have a strong focus on developing health promotion interventions. Many of the research projects in Denmark are supported by strong collaborations with field leading international researchers.

**Methods:**

Interest and utilization of the concept has also undergone a prodigious development in Denmark from a practice and policy perspective. The national sport for all organisation, DGI, has incorporated PL as one of their key concepts which guides all their activities. The national school sports organisation, Danish School Sports, has also adopted the concept and have currently funded an industrial PhD project, which investigates health-related correlates to PL while also spreading knowledge about the concept in the organisation and in schools. In 2018, a national cross-sectoral network for PL interested parties was established (https://www.pl-net.dk).

**Results and discussion:**

This presentation will focus on providing a short overview and discussion of the research, practice and policy development in Denmark while exemplifying this development by presenting the recent interest from several large-scale school-based intervention projects in more detail.

